# Beyond *SGCE*: expanding the clinical and molecular spectrum of *KCTD17*- and *KCNN2*-related myoclonus-dystonia

**DOI:** 10.3389/fneur.2026.1728361

**Published:** 2026-03-30

**Authors:** Magdalena Krygier, Emilia J. Sitek, Magdalena Chylińska, Szymon Ziętkiewicz, Marta Zawadzka, Jarosław Dulski, Michał Schinwelski, Grażyna Kostrzewa, Jolanta Wierzba, Rafał Płoski, Michael Zech, Maria Mazurkiewicz-Bełdzińska

**Affiliations:** 1 Department of Developmental Neurology, Medical University of Gdańsk, Gdańsk, Poland; 2 Laboratory of Clinical Neuropsychology, Neurolinguistics and Neuropsychotherapy, Division of Neurological and Psychiatric Nursing, Faculty of Health Sciences, Medical University of Gdańsk, Gdańsk, Poland; 3 Department of Neurology, St. Adalbert Hospital, Copernicus PL Ltd., Gdansk, Poland; 4 Department of Adult Neurology, Medical University of Gdańsk, Gdańsk, Poland; 5 Intercollegiate Faculty of Biotechnology, University of Gdańsk, Gdańsk, Poland; 6 Department of Neurology, Neurodegenerative Diseases and Neuroimmunology, Faculty of Health Sciences, Medical University of Gdańsk, Gdańsk, Poland; 7 Division of Neurological and Psychiatric Nursing, Faculty of Health Sciences, Medical University of Gdańsk, Gdańsk, Poland; 8 Neurocentrum-Miwomed, Neurological Clinic, Gdańsk, Poland; 9 Department of Medical Genetics, Medical University of Warsaw, Warsaw, Poland; 10 Department of Internal and Pediatric Nursing, Institute of Nursing and Midwifery, Medical University of Gdańsk, Gdańsk, Poland; 11 Institute of Human Genetics, School of Medicine and Health, Technical University of Munich, Munich, Germany; 12 Institute of Neurogenomics, Helmholtz Zentrum München, Munich, Germany; 13 Institute for Advanced Study, Technical University of Munich, Garching, Germany

**Keywords:** dystonia, KCNN2, KCTD17, memory impairment, myoclonus, neuropsychiatric symptoms, pediatric movement disorders, SGCE

## Abstract

Myoclonus-dystonia syndrome (MDS) is a clinically and genetically heterogeneous movement disorder characterized by myoclonus and dystonia as its core features. While mutations in the *epsilon-*sarcoglycan gene (SGCE) account for most familial cases, heterozygous pathogenic variants in *KCTD17* and *KCNN2* have recently been described as novel genetic causes of MDS. *W*e describe three patients from two Polish families presenting with progressive movement disorder combined with other features. Exome sequencing (ES) identified a novel heterozygous *KCTD17* variant c.461 T > A, p.(Met154Lys) in a five-year-old girl with abnormal gait, postural instability, myoclonus, and tongue dyskinesia. In a 38-year-old woman and her 17-year-old daughter, both showing tremor, myoclonus, dystonia, and psychiatric symptoms, ES detected a heterozygous canonical splice-site c.1780-2A > G variant in *KCNN2*. Neuropsychological evaluation suggested a gene-specific effect of *KCNN2* on psychiatric and cognitive functioning, including significant episodic memory impairment. This study broadens the clinical and molecular spectrum of *KCTD17*- and *KCNN2*-related MDS and highlights distinctive features compared with *SGCE*-MDS, focusing on disease progression, treatment response, and neuropsychiatric involvement. Recognition of these patterns may guide molecular diagnosis and the management of specific MDS types.

## Introduction

Myoclonus-dystonia syndrome (MDS) is a rare movement disorder characterized by the presence of myoclonus and dystonia as the core features. MDS is clinically and genetically heterogeneous, with several causal genes identified so far (1). Heterozygous variants in *SGCE*, encoding the epsilon member of the sarcoglycan family, account for the majority of familial MDS (2, 3). *SGCE* myoclonus-dystonia typically presents in the first decade of life with non-epileptic myoclonic jerks, affecting predominantly proximal muscles of the upper limbs, trunk, and neck. Over half of the adult patients also present mild to moderate upper body focal or segmental dystonia (4). Importantly, affected children may display a distinct dystonic pattern, characterized by action-specific lower-limb dystonia triggered by walking or running (5, 6). The hallmark of *SGCE*-MDS is the amelioration of motor symptoms with alcohol. The inheritance is autosomal dominant, with reduced penetrance of maternally derived *SGCE* alleles, caused by maternal imprinting (7).

Although *SGCE* is a major disease gene for myoclonus-dystonia, a substantial number of patients, varying from 20 to 80% depending on the studied cohort, test negative for the *SGCE* mutations (6). The co-occurrence of myoclonus and dystonia is observed in other combined dystonia syndromes, such as *ADCY5*-, *ANO3-*, or *NKX2-1*-related diseases (1). Moreover, several other pediatric movement disorders, including inherited defects of dopamine metabolism, can mimic MDS, but their phenotype is usually distinct or broader from classical MDS (8). In the Online Mendelian Inheritance in Man (OMIM) database, only three genes, namely *SGCE*, *KCTD17*, and *KCNN2*, have currently been linked to “myoclonic dystonia.” However, recently described phenotypes, such as *NUS1*-associated movement disorders, may expand this spectrum in the future (9, 10).

In 2015, Mencacci et al. identified a heterozygous missense *KCTD17* variant c.434G > A, p.(Arg145His) in two unrelated families of British and German descent affected by MDS (11). Since this initial report, only a few additional cases of *KCTD17*-MDS have been documented globally (12–15). Similarly, to date, no more than seven families have been reported with *KCNN2*-MDS (16–22). These observations highlight the need for further studies to better characterize the clinical spectrum of these ultrarare disorders, as well as to identify the most effective therapeutic options.

## Subjects and methods

### Participants

We describe three previously unreported individuals from two Polish families with variants in *KCNN2* (patient P1 [P1] and P2, family A) or *KCTD17* (P3, family B). The families were referred to the tertiary Developmental and Adult Neurology Departments at the Medical University of Gdansk (Poland) due to progressive movement disorder combined with other features. Clinical assessments involved a retrospective analysis of medical records, a detailed medical interview, and prospective neurological examinations with video recording. Brain magnetic resonance imaging (MRI) was available for all subjects. Electrophysiological studies in P1 and P3, including a nerve conduction study (NCS), F-wave study and electromyography (EMG), were performed on a Dantec Keypoint G4 EMG / NCS / EP Workstation, according to the protocol recommended by the American Association of Neuromuscular and Electrodiagnostic Medicine (23, 24).

Neuropsychological assessment of P1 and P2 (family A) comprised of general intellectual functioning testing, cognitive screening, manual dexterity and praxis, language and visuospatial function, working memory and processing speed, calculation, episodic memory and learning as well as executive function assessment. Most of the cognitive tasks were administered to both P1 and P2. Additionally, mood was assessed with Beck Depression Inventory-II (BDI-II; Supplementary Table S1).

The study adheres to the principles set out in the Declaration of Helsinki. Written informed consent for genetic testing and for publishing genetic and clinical data, including videos and photographs was obtained from patients (P1, P2) or their parents (P1, P3).

### Exome sequencing and data analysis

In family A, DNA from the patient and the parents was prepared for exome sequencing using the Twist Human Exome 2.0 Plus (1) Comprehensive Exome Spike-in and (2) Mitochondrial Panel Kit and sequenced as 100 bp paired-end reads on a NovaSeq6000 (Illumina Inc.) to average 71-fold coverage, with >97% of target sequences covered >20-fold. After preprocessing of the sequencing data using Illumina bcl2fastq, adapter sequences were removed using the *cutadapt* tool. The Human Genome Assembly GRCh37 (hg19) was used for alignment with *Burrows-Wheeler Aligner* (BWA) tool. PCR duplicates and optical duplicates were removed using the *Picard MarkDuplicates* tool. Variant calling was performed using several software tools: *Genome Analysis Toolkit 4* (GATK 4.2.3.0) and *samtools* for single nucleotide variants (SNVs), *Pindel* for indels up to 20 bp, and *ExomeDepth* for copy number variations (CNVs). Filtering and prioritization of variants was done with the in-house software EVAdb^1^ focusing on rare nonsynonymous variants affecting preselected genes know to be associated with the clinical features of the affected individuals.

In family B, exome sequencing was performed only for the proband using genomic DNA isolated from peripheral blood and the Twist Human Core Exome 2.0 + Comp Spike-in + Twist mtDNA Panel (Twist Bioscience, United States), according to the manufacturer’s protocol. Raw sequencing data were analyzed as previously described (25). In silico pathogenicity prediction of variants was carried out using the in-house developed GeneBe platform in combination with Varsome data (26, 27). Selected variants were further analyzed by deep amplicon sequencing of PCR products using the NEXTERA XT kit (Illumina, United States) on an Illumina NGS platform in the proband and her parents (family segregation analysis).

### Protein structural modelling and analysis

The pentameric model of KCTD17 (region 19 to 190) was constructed by SWISS-MODEL (accession date 16.01.2025), based on the 3DRX crystal structure of KCDT5 (28, 29). The model-template identity was 79.07%. A M154K mutation was introduced into one of the subunits and so modified system was prepared for Molecular Dynamics (MD) simulation by CHARMM-GUI (30). After energy minimization and equilibration, MD simulation was conducted for 200 ns (GROMACS 2025.1) (31). Resulting trajectory was visualized and analyzed by VMD 1.9.4a55 (32).

## Results

### Family A with a canonical splice-site variant in KCNN2

#### Neurological symptoms

**Figure 1:**
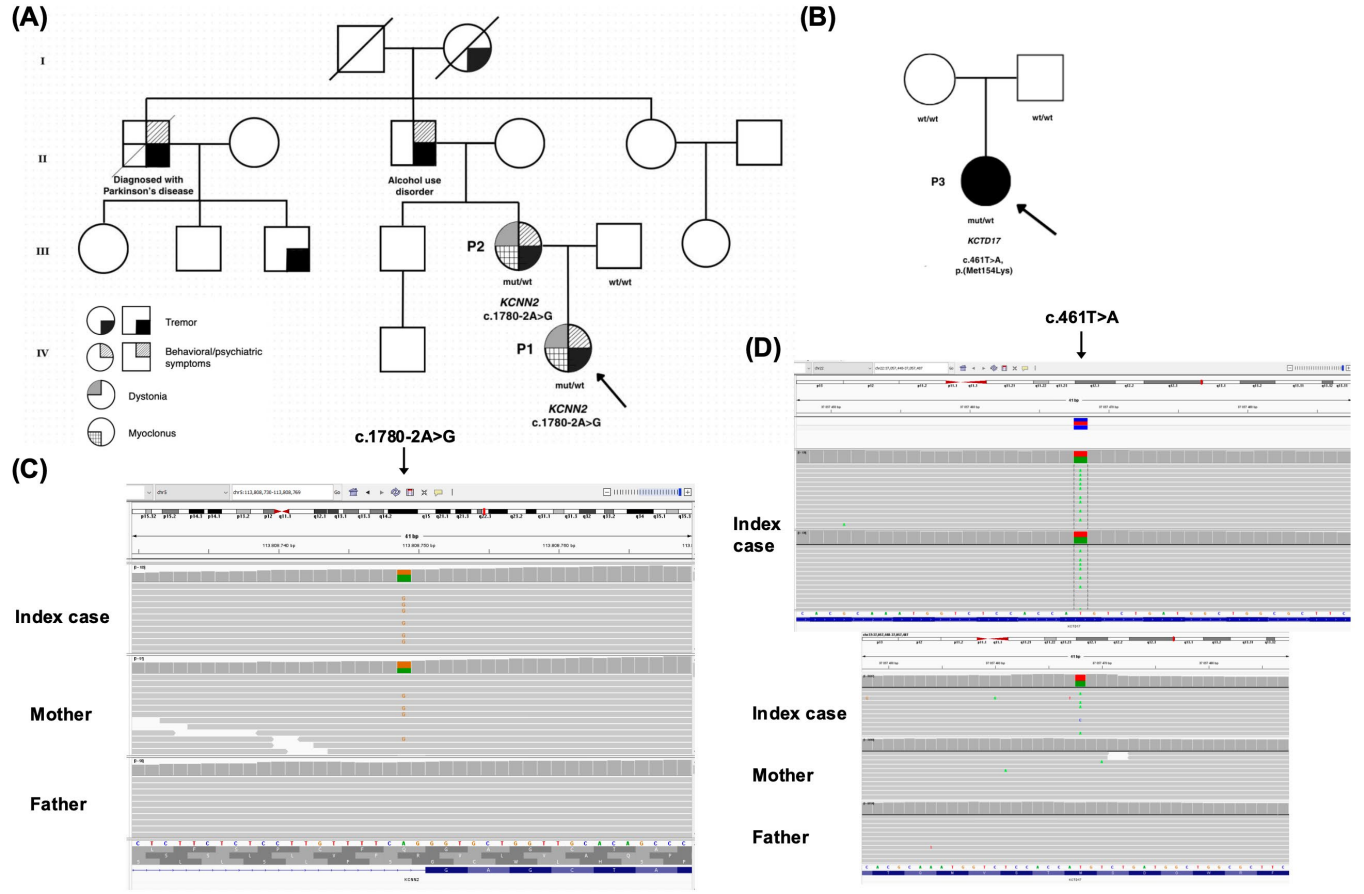
Pedigree drawings and genetic results for families with *KCNN2* and *KCTD17* variants. **(A)** Family A with patient 1 (P1) and patient 2 (P2). **(B)** Family B with P3. Index patients are indicated by arrows. Mut/wt denotes the reported *KCNN2* or *KCTD17* variants in the heterozygous state; wt/wt denotes a homozygous wild-type allele. **(C)** Trio exome sequencing results for Family A. **(D)** Exome sequencing results for P3 and amplicon deep sequencing results for Family B.

P1 is a 17-year-old woman born after an uneventful pregnancy and delivery with normal birth parameters. Her mother (P2) is similarly affected. Several maternal relatives also displayed tremor, psychiatric and behavioral symptoms, and/or a history of psychiatric treatment, although detailed clinical data were not available (Figure 1A). The patient’s early motor milestones were mildly delayed, with independent sitting at 10 months and walking at 18 months. A hand tremor during manual tasks (eating, holding objects, drawing) was noted from age three, later involving the trunk and head, and accompanied by balance and coordination difficulties. During school years, she developed abnormal hand posturing during writing, consistent with action dystonia. At age 17, on examination she had mild dysarthria, tongue and head tremor, upper limb postural and action tremor, action-induced dystonia during writing, and myoclonus predominantly affecting the hands and feet, with lesser involvement of the arms and neck. In addition, mild ataxia was present, with a wide-based gait and dysmetria on finger–nose and heel–shin testing (Supplementary Video 1).

In P2, the 37-year-old mother of P1, upper limb and head tremor, jerks during writing and overall motor slowing were first noticed at around 7 years of age. Over time, the symptoms worsened and impacted school and daily activities significantly. From her 30s, she experienced generalized muscle stiffness and involuntary muscle contractions of the hands and feet, triggered by writing and walking, though symptoms were considered mild and not disabling. At age 33, on examination she had mild dysarthria, jerky tremor of the head, upper limb postural and action tremor, superimposed with multifocal myoclonus, and bilateral hand dystonia more pronounced on the right. No ataxia was seen. At the follow-up examination at age 38, her neurological status remained stable, characterized by predominant postural and action tremor of the head and hands, accompanied by myoclonus and hand posturing (Supplementary Video 2). Both P1 and P2 reported exacerbation of symptoms with emotions, fatigue, and caffeine. Laboratory investigations, brain MRI and video-electroencephalography (EEG) in P1 and P2 were unremarkable.

In P2, pharmacological treatment over the years included propranolol (up to 80 mg/day), biperiden (up to 12 mg/day), levetiracetam, clonazepam and primidone (up to 250 mg/day). The most notable, though only partial, symptomatic improvement was achieved with the anticholinergic agent biperiden, while propranolol and primidone showed no effect. The patient reported initial amelioration of involuntary movements with alcohol, however, this effect is no longer observed.

#### Neuropsychiatric symptoms

Both P1 and P2 have a history of psychiatric symptoms since childhood. In P1, social anxiety disorder and obsessive-compulsive behaviors were present from primary school. At age 13, she was hospitalized for anorexia nervosa. At the age of 14 psychological assessment revealed intelligence within the normal range with impaired visual perception and memory, slowed speech and writing, as well as regression in calculation skills. During adolescence, repeated psychiatric evaluations indicated mixed behavioral and emotional disorder, severe emotional dysregulation with mood lability and irritability (including aggressive behavior), and school phobia. Due to her rather poor social engagement autism spectrum disorder (ASD) was suspected, but the psychological assessment did not confirm it. She could recognize and express emotions. On the recent neuropsychological testing (Supplementary Table S1), P1 showed selective cognitive deficits, particularly in episodic memory and learning (both verbal and visual) and executive functions (initiation, planning, organization). She presented also with slowed processing speed and impaired working memory. Visuomotor coordination and manual dexterity were significantly affected. Psychotic symptoms (mild visual, olfactory, and tactile hallucinations) and food fads were also noted.

P2 has a history of obsessive-compulsive disorder (OCD) since childhood and psychotic symptoms beginning in early adulthood. She has been hospitalized multiple times in psychiatric wards for behavioral and psychotic symptoms and was formally diagnosed with paranoid schizophrenia. Her history also includes alcohol abuse and four suicide attempts. Neuropsychological evaluation at age 38 revealed a generalized cognitive decline, mostly pronounced in memory and executive functions. Overall intellectual functioning appeared below average or borderline. Executive dysfunction was consistent with the diagnosis of schizophrenia, with impairments in planning, task control, and strategy shifting. However, some of these deficits—especially impaired set-shifting and perseveration—are also typical for basal ganglia dysfunction. Episodic memory dysfunction suggested bilateral hippocampal involvement. Cognitive deficits were accompanied by depressed mood (Supplementary Table S1). In comparison to her daughter (P1) deterioration in manual dexterity, visual memory and executive function was more prominent.

#### Electrophysiological study in P1

A nerve conduction study revealed normal conduction parameters in the nerves of the upper and lower extremities. Resting EMG of the left first dorsal interosseous muscle demonstrated abnormal spontaneous activity—irregular (myoclonic) bursts of approximately 100 ms and longer (dystonic) bursts up to 200 ms in duration, with a frequency of about 50 Hz (Supplementary Figure S1A). Moreover, in P1, a high-frequency tremor (approximately 16 Hz) was observed (Supplementary Figure S1B). Motor unit action potentials (MUAPs) showed normal parameters of duration, but increased amplitude.

#### KCNN2 c.1780-2A > G

Trio-based ES identified a heterozygous splice-site c.1780-2A > G variant in *KCNN2* (NM_021614.4) in P1 and P2 (Figures 1A,C). The canonical splice-site variant c.1780-2A > G is predicted to result in the loss of an acceptor splice site of exon 5 (NM_021614.4) of *KCNN2* and is classified as likely pathogenic. The variant is not listed in ClinVar, however, another canonical splice-site variant of *KCNN2* exon 5, affecting the donor splice site (c.1890 + 2 T > C), is reported as likely pathogenic (2 independent submitters, variation ID: 933148).

### Family B with a *de novo* missense variant in KCTD17

P3 is a 5-year-old girl with unremarkable perinatal and family history, initially referred due to muscular hypotonia and motor developmental delay. She started physiotherapy at 3 months for hypotonia and began sitting independently at 9 months and walking by 18 months. Her gait has been unstable from the beginning, with a broader base, hyperextension of the trunk, frequent falls, and coordination problems. In addition, she had involuntary movements of the neck, trunk, and limbs, which contributed to balance problems and falls. At 5 years, on examination, she had an unsteady gait and posture, impaired by generalized hyperkinesia, mild muscular hypotonia, hyperlordosis, left hand posturing, and subtle myoclonic jerks of the trunk, neck, and arms. Her speech was dysarthric and hypophonic with associated tongue dyskinesia. No ataxia was observed (Supplementary Video 3).

Psychological evaluation in P3 showed normal intellectual development. Cognitive processes such as perception, attention, memory, and thinking were age-appropriate. Her speech development and vocabulary were adequate, but articulation was impaired by mixed dysarthria and dysphonia. On manual tasks, she had incorrect pencil grip. Overall, her gross and fine motor abilities were described as significantly impaired. Brain MRI and video-EEG showed no abnormalities.

#### Electrophysiological study in P3

In the P3 subject, EMG of the right deltoid muscle was performed. At rest, the EMG revealed irregular bursts (approximately 7–8 Hz) with durations of 45–80 ms and spike frequencies of about 28 Hz, associated with clinical myoclonus (Supplementary Figure S1C). The parameters of MUAPs, including amplitude and duration, were within the normal range. In both families, the absence of associated EEG abnormalities and burst durations ≥50 ms are consistent with subcortical myoclonus.

#### KCTD17 c.461 T > A, p.(Met154Lys)

In P3, exome sequencing (ES) identified a heterozygous c.461 T > A, p.(Met154Lys) variant in KCTD17 (NM_001282684.2). Deep amplicon sequencing excluded the presence of this variant in both parents (Figures 1B,D). According to ACMG criteria, the variant was classified as likely pathogenic (8 points - when the *de novo* status was considered). Bioinformatic prediction supported this classification (CCRS 99.12; AlphaMissense 1.0; phyloP100 7.96; REVEL 0.79; CADD 26). The variant was not reported in the GnomAD database (v. 4.1.0) nor in ClinVar (v. 07-Jul-2025).

#### Protein structural modelling and analysis of the KCTD17 variant

**Figure 2:**
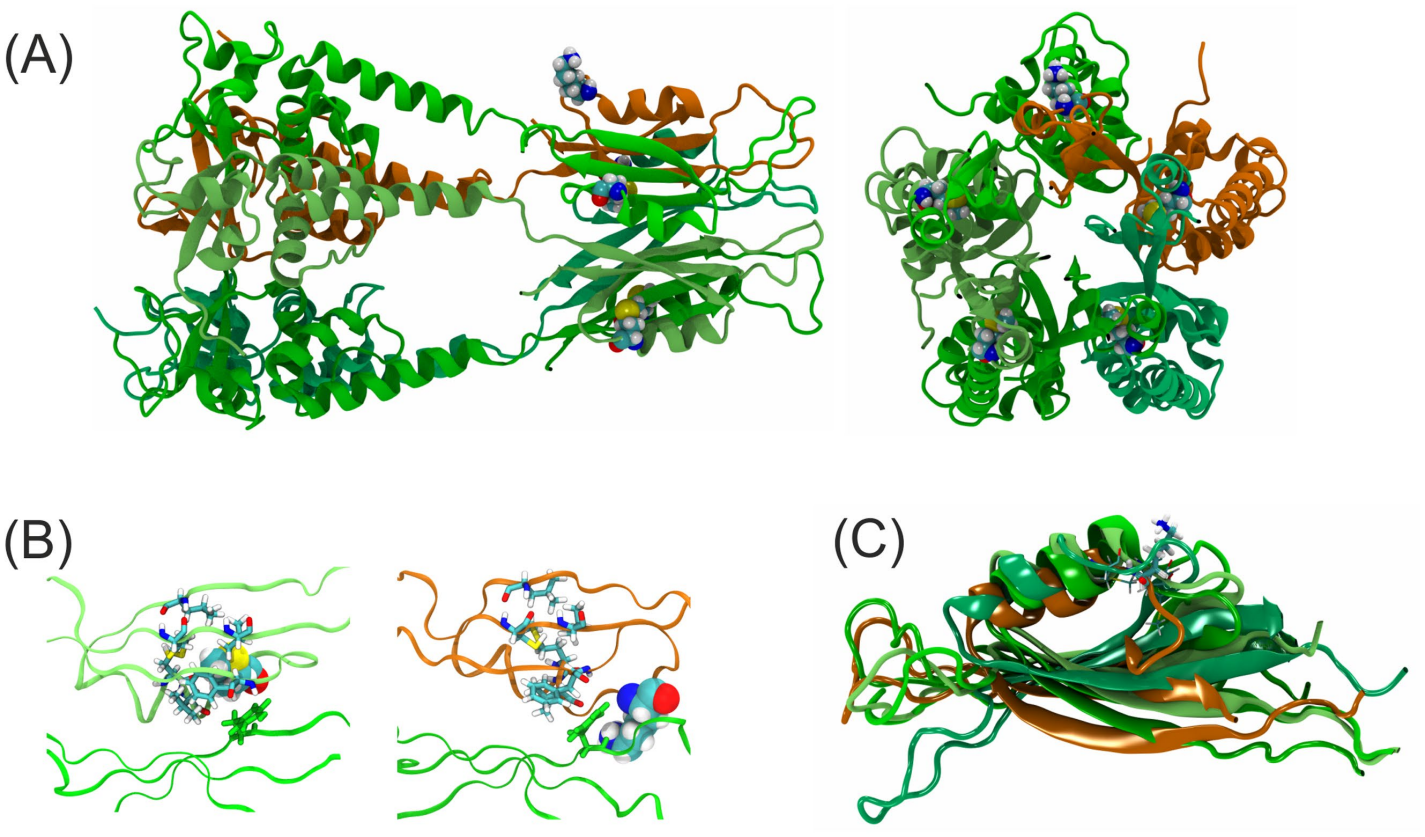
Protein structural modeling and analysis of the *KCTD17* p.(Met154Lys) variant. **(A)** Homology model of KCTD17 pentamer (residues 19–190), final frame from 200 ns MD trajectory. Subunit with M154K mutation marked in orange, native subunits marked with shades of green. Residue 154 (K in orange subunit, M in green subunits) in spacefill representation. Left, view perpendicular to the pentamer axis, right, view along the pentamer axis. **(B)** Molecular surroundings of the residue 154. A representative frame from MD trajectory. Depicted are two adjacent subunits in ribbon representation, with residue 154 in one of them in spacefill representation and the residues making contacts with residue 154 in stick representation. The thyrosine 137 of the neighboring subunit marked green. Left, both subunits WT, right—top subunit bearing M154K mutation, lower subunit WT. **(C)** Superposition of all subunits (for clarity, presented fragment 131–188 only; fitted over fragment depicted), showing typical behavior of residue 154 vincinity (a representative frame from MD trajectory shown). A mutation-bearing protomer marked in orange, WT subunits in the shades of green. In stick representation, residues 154 form all subunits are marked, with K154 in thicker lines.

A homology model of a fragment of KCTD17 was constructed, assuming its pentameric arrangement (33). The locus of the mutation on the protein structure is depicted in Figure 2A.

The residure 154 in the WT structure is a large, hydrophobic methionine residue, placed in a structured domain just after the N-terminal BTB domain and a short linker. The residue 154 is positioned at the beginning of a loop fragment joining an alpha-helical fragment with a strand of a beta sheet. In all four WT subunits of a modeled pentamer, this residue is buried inside this domain in a hydrophobic pocket, close to the intersubunit interface. The Met154 makes contacts with residues M150, V151 (on the alpha helix), and L140, F160, C182, V184 (on the beta sheet) of the same protomer, and with T137 of the neighboring one (Figure 2B). As expected from the change of physico-chemical properties, lysine presented in 154 position of mutated subunit was seen replaced to the outside of the hydrophobic pocked and remained expelled from that position for the whole 200 ns of MD trajectory (while in all other WT subunits, methionines 154 remained stably buried, except for a single event in the subunit neighboring to the mutated one, not shown). Apart from the clear change of conformation of 154 residue, the structural influence of the mutation is visible also in the adjacent regions. The superposition of all simulated subunits (Figure 2C) reveals that during the simulation, particular WT subunits retain very similar conformation, while the M154K subunit tends to reposition the loop between its preceding helix and subsequent beta strand, in some moments of the trajectory even disturbing the arrangement of the first residues of a beta strand.

While the exact molecular mechanism leading from the conformational changes described to the phenotype observed remains to be detailed by more extensive structural research backed with *in vitro* functional studies, it may well be speculated from their magnitude that the presence of one or more mutated subunits in the same pentamer in a heterozygous patient may well lead to the disturbance of oligomerization, overall stability, allosteric signaling or a partner binding—explaining the negative dominant effect.

## Discussion

Myoclonus-dystonia is a heterogeneous clinical syndrome, and considerable efforts have been made to identify novel genes associated with this unique phenotype. Despite overlapping motor features, the *KCTD17*- and *KCNN2-*MDS show distinct characteristics (as summarized in Table 1), the recognition of which is crucial for accurate diagnosis, appropriate treatment, identification of comorbidities, and genetic counseling.

**Table 1:** Clinical and molecular characteristics of myoclonus-dystonia syndromes caused by *SGCE*, *KCTD17*, and *KCNN2* variants.

MDS Gene	SGCE	*KCTD17*	*KCNN2*
Age at onset of dystonia or myoclonus	First decade (mean 6 years; range 6 months–80 years)	First decade (range 4–51 years)	First decade (range 3–12 years)
Primary movement symptom	Myoclonus, less commonly dystonia	Myoclonus or jerky tremor, less commonly dystonia	Tremor with superimposed myoclonus, rarely dystonia
Body distribution of myoclonus	Neck, trunk, and upper limbs (proximal> distal)*; “lightning-like” or “tic-tac” jerks	Upper limbs (proximal> distal), neck, face*	Upper limbs (distal>proximal), neck, face*; sometimes occurring in a “shivering-like” fashion
Dystonia	In over half of patients	In allmost all patients (12/13**)	In almost all MDS patients (11/12 individuals from seven families), but other overlapping motor phenotypes without dystonia also described
Body distribution of dystonia	Neck, upper limbs (cervical dystonia, writer’s cramp, hand posturing); lower limb action dystonia (more common in children)	Cranio- cervical region (including oromandibular dystonia, laryngeal dystonia, and blepharospasm) and upper limbs; can progress to generalized dystonia	Neck, upper limbs (cervical dystonia, writer’s cramp, hand posturing)
Progression of symptoms	Myoclonus causing significant functional disability; stable mild to moderate dystonia in most cases; possible spontaneous remission of limb dystonia during childhood or adolescence	Increasing severity of dystonia spreading from the cranio-cervical region or upper limb to other sites; mild myoclonus	Gradual progression of motor symptoms, with later stabilization in some patients; disabling myoclonus and tremor; mild to moderate dystonia
Improvement with alcohol	Yes	No	Early in the disease course, not sustained later in life
Eliciting and aggravating factors	Posture, action, stress, caffeine, touch and acoustic stimuli	Posture, action, stress	Posture, action, touch and acoustic stimuli, stress, caffeine (in two families), fatique
Neuropsychiatric symptoms	Yes, common (in about 65% of cases in a large cohort study)	Rare (2/13**)	Yes, prominent and variable
Cognitive dysfunction	No	No (low normal cognitive abilities in one case)	Yes, common
Additional features	Postural tremor of the upper limbs, seizures (rare)	Infantile hypotonia, motor delay, unsteady gait	High-frequency, small-amplitude hand tremor (resting and action), head tremor, mild cerebellar ataxia, opsoclonus, psychomotor delay, bordeline/mild intellectual disability***
GPi DBS response	Good (significant and sustained improvement in dystonia, myoclonus, and quality of life)	Good (improvement in myoclonus and dystonia, including cranial and laryngeal dystonia), but limited data on long-term outcomes	Unknown (limited and contradictory data)
Reported disease-associated variants	Various variants reported (> 100): nonsense, missense, frameshift, splice-site, exon deletions, whole-gene deletions	Missense and splice-site variants#: c.434G > A, p.(Arg145His); c.461 T > A, (p. Met154Lys); c.785C > T, (p. Ser262Phe)##; c.229C > A, (p. Leu77Ile); c.508-2A > T; c.508-1G > C	Missense, splice-site, and nonsense variants##: c.1112G > A, p.(Gly371Glu); c.862_863delinsTC, p.(Ile288Ser); c.1831C > A, p.(Leu611Ile); c.1709C > T, p.(Ser570Phe); c.1084G > A; c.583C > T, p.(Gln195Ter); c.1780-2A > G
Genotype–phenotype correlations	Not identified	So far not reported (limited data avaialble)	So far not reported (limited data avaialble)
References	(3–8, 37)	(11–15)	(16–22)

*Can affect all body parts; **Molecularly confirmed cases; ***Variable motor and non-motor features described in association with other *KCNN2*-related phenotypes; #Additional missense and truncating variants detected in association with other *KCNN2*-related phenotypes; ##Variant detected in asymptomatic mother; GPi DBS, Globus Pallidus internus Deep Brain Stimulation.

### KCNN2-MDS

*KCNN2* encodes the small-conductance calcium-activated potassium channel 2 (SK2), a member of the voltage-independent potassium channel subfamily regulated by intracellular calcium. SK1–3 channels are broadly expressed in the brain and play a role in the regulation of neuronal excitability, repetitive neuronal firing, synaptic transmission, and synaptic plasticity (20, 34).

In 2020, Balint *et* al. reported a novel missense *KCNN2* variant c.1112G > A, p.(Gly371Glu) in a large British family with autosomal-dominant tremulous myoclonus-dystonia (16). Affected family members had childhood-onset dystonia, mainly involving the hands and neck, accompanied by a fast tremor with superimposed myoclonus and, in some patients, mild cerebellar signs. Subsequently, Mochel et al. described 11 individuals, nine of whom carried *de novo KCNN2* variants, thereby broadening the clinical spectrum of *KCNN2*-related phenotypes to include developmental delay (DD), intellectual disability (ID), ASD, psychiatric symptoms, epilepsy, tremor, myoclonus-dystonia, cerebellar ataxia, chorea, and parkinsonism (20).

Based on these reports and further case descriptions, a heterogeneous clinical phenotype becomes apparent (16–22). In contrast to other MDS, psychomotor delay or intellectual disability/cognitive dysfunction occur in most cases. The core motor feature, though not universally present, is high-frequency small-amplitude hand tremor, present mostly on posture and action, and typically beginning in childhood. Superimposed on this are myoclonic jerks, which can affect all body parts, but are mostly pronounced in the hands, arms, neck, and trunk. *KCNN2* variants present with ‘shivering-like’ distal predominant tremulous myoclonus in contrast to the ‘lightning-like’ proximal jerks seen in *SGCE* patients. Myoclonus may or may not be stimulus-sensitive, is commonly exacerbated by emotions, and cause significant functional impairment. Dystonia is less frequent, and when present, tends to be mild to moderate, manifesting as torticollis, dystonic hand posturing, or writer’s cramp. Of note, even when dystonia is absent on clinical examination, EMG patterns resemble those seen in myoclonus-dystonia (19). Signs of mild cerebellar dysfunction, such as broken pursuit, hypermetric saccades, rotatory end-gaze nystagmus, limb dysmetria, and impaired tandem gait, have been documented in several families and, when present, should prompt suspicion of *KCNN2*-MDS.

So far, oral medications have shown limited benefit in managing motor symptoms in *KCNN2*-MDS. Importantly, the use of some anti-seizure drugs may be restricted due to co-occurring psychiatric symptoms. In a case reported by Pauly et al., treatment with piracetam, propranolol, valproic acid, primidone, oxitriptan, sodium oxybate, and levodopa was ineffective or led to intolerable side effects. While levetiracetam and clonazepam did reduce myoclonus, they might exacerbate irritability and aggressive behavior. Zonisamide has significantly reduced myoclonus in one case and may be an effective therapeutic option (21). Although alcohol may provide initial amelioration of symptoms, this effect is not sustained later in life.

To date, bilateral deep brain stimulation (DBS) of the globus pallidus internus (GPi) has been reported in only two patients with *KCNN2*-MDS. The case described by Pauly et al. did not show sustained benefit from GPi-DBS despite extensive reprogramming (21). In contrast, a 38-year-old patient reported by Stodulska et al. demonstrated marked improvement of cervical and upper limb dystonia, along with resolution of myoclonus in these body sites during a 24-month follow-up (22). While these observations are promising, further studies are needed to evaluate the therapeutic potential of DBS in *KCNN2*-related myoclonus-dystonia.

### KCTD17-MDS

*KCTD17* (MIM 616398) encodes a substrate adaptor protein for the Cullin3-based E3 ubiquitin ligase complex, of unknown precise biological function. *KCTD17* has been implicated in ciliogenesis and the regulation of calcium ion homeostasis. *KCTD17* is highly expressed in the basal ganglia and may play a role in postsynaptic dopaminergic signaling (11, 35).

*KCTD17*-related myoclonus-dystonia is characterized by the presence of dystonia predominating over myoclonus. Motor symptoms typically manifest between early childhood and early adulthood, although onset in the sixth decade has also been reported (14). Our observations confirm that infantile hypotonia and delayed motor development are important early features (12, 13). Difficulties such as unsteady gait, poor coordination, and frequent falls are likely attributable to both hypotonia and mild proximal myoclonic jerks. Myoclonus is most pronounced in the upper body (arms, neck, face), action-aggravated, and frequently associated with tremor, described as jerky tremor. Dystonia is present in nearly all affected individuals. It often manifests as jerky or tremulous dystonia and predominantly affects the upper limbs and cranio-cervical region, with laryngeal dystonia commonly reported. Although laryngeal involvement was first described in older individuals (>60 years), subsequent reports showed that oro-lingual-laryngeal dystonia may occur already in childhood (16). Myoclonus remains relatively mild, whereas dystonia follows a progressive course, gradually spreading to other body sites.

There is limited information available on the treatment of *KCTD17*-MDS. In a case reported by Marcé-Grau et al., several oral medications, such as clonazepam, L-dopa, tetrabenazine, zonisamide, pimozide, and trihexyphenidyl, were found to be ineffective (16). The outcomes of GPi DBS have been described in two patients, each demonstrating marked improvement in dystonia (including orolingual dyskinesia and speech) and myoclonus (11, 13). Therefore, GPi DBS is currently the most effective treatment, although evidence regarding long-term outcomes is still scarce. So far, none of the patients reported alcohol-responsiveness.

### Psychiatric and cognitive symptoms in myoclonus-dystonia syndromes

Non-motor symptoms are an integral feature of different dystonia phenotypes. In hereditary dystonia, anxiety, depression, and OCD are among the most common features, but the spectrum of abnormalities extends beyond purely psychiatric disorders (36, 37). Depression and anxiety are consistently reported across multiple studies, suggesting potential shared mechanistic pathways, although the underlying pathogenesis is still poorly understood (38, 39). Cognitive profiles in dystonia are variable yet overlapping patterns of impairment have been observed. Overall, cognitive deficits are more pronounced in genetic than acquired forms of dystonia, but the longitudinal neuropsychological observations are limited (40). Importantly, studies on genetically defined dystonia suggest gene-specific effects on psychiatric and cognitive manifestations.

Our report highlights the significant role of psychiatric disorders in individuals with *KCNN2*-MDS, who may present with a broad spectrum of symptoms, including OCD, anxiety, depression, eating disorders, attention-deficit/hyperactivity disorder (ADHD), substance use disorders, and psychotic disorders, including schizophrenia. These manifestations can appear as early as in childhood, are often resistant to standard therapies, and may substantially impair daily functioning or even become life-threatening.

SK2 channels modulate neuronal excitability by causing membrane hyperpolarization and have a wide range of functions in CA1 hippocampal neurons, which are a very important element of memory circuitry (41). Both P1 and P2 demonstrated marked and generalized episodic memory impairment. Difficulties in delayed recall could not be attributed to executive deficits as the patients had impaired spontaneous retrieval, cued recall and recognition. Also, they cannot be explained by fatigue as both patients had increasing learning curves. Of note, these prominent episodic memory deficits, much more severe than typically observed in patients with dystonia and in most basal ganglia disorders, were not material-dependent as they appeared in both verbal and visual task in the two subjects. Thus, this severe memory impairment is very likely to stem from primary hippocampal dysfunction in CA1 region. The reduced volume of CA1 is related to poor long-term retrieval not only in older adults, but also in individuals with schizophrenia (42, 43). So far, the pattern or severity of memory impairment in *KCNN2*-MDS has not been described in detail, so it is difficult to compare our findings on memory deficit against other case descriptions. Patients with *KCNN2* mutations should undergo extensive memory testing as a part of neuropsychological assessment. The assessment needs to address both spontaneous recall and recognition to prove primary memory deficit.

To date, psychiatric manifestations have been reported in two members of a single family with *KCTD17*-MDS (11). A 44-year-old woman presented with anxiety and social phobia, while her 35-year-old relative exhibited obsessive traits and depression. Neither intellectual disability nor cognitive decline has been documented, although an 8-year-old patient had cognitive abilities in the low-normal range (12). Given the limited data available, it remains uncertain whether *KCTD17*-MDS is consistently associated with cognitive or neuropsychiatric impairments. Extended follow-up and detailed neuropsychological evaluations will be crucial in addressing this issue.

## Conclusion

Our findings broaden the clinical and genetic spectrum of rare *KCTD17*- and *KCNN2*-related myoclonus-dystonia. Distinct clinical profiles, including neurodevelopmental, motor, neuropsychiatric, and cognitive features, emphasize the importance of comprehensive clinical evaluation of MDS patients. Moreover, advancing our understanding of disease progression and treatment responsiveness is essential for developing management strategies for specific MDS subtypes.

## Data Availability

The original contributions presented in the study are publicly available. This data can be found here: https://www.ncbi.nlm.nih.gov/clinvar/ with the following accessions SUB16050892, SUB16050893.

## Footnotes

1
github.com/mri-ihg/EVAdb

## Supplementary material

SUPPLEMENTARY VIDEO 1 Patient 1 (P1, age 17) with *KCNN2-*MDS presenting high-frequency postural tremor of the hands with superimposed hand myoclonus, gait and limb ataxia, and action dystonia during writing.

SUPPLEMENTARY VIDEO 2 Neurological examination of the Patient 2 (P2, age 38) with *KCNN2*-MDS demonstrating postural and action tremor of the head and hands, accompanied by myoclonus and hand posturing. No signs of ataxia or gait disturbance were observed.

SUPPLEMENTARY VIDEO 3 Patient 3 (P3, age 5) with *KCTD17*-MDS showing an unsteady gait and posture, impaired by generalized hyperkinesia, mild left hand posturing, and subtle myoclonic jerks of the arms. No ataxia was seen.
