# Supplementary material for: Beyond SGCE: expanding the clinical and molecular spectrum of KCTD17- and KCNN2-related myoclonus-dystonia
Source: Front Neurol. 2026 Mar 30;17:1728361. doi: 10.3389/fneur.2026.1728361 (PMC13070805; doi:10.3389/fneur.2026.1728361)

**Supplemental data**

Table S1. Neuropsychological assessment results of mother and daughter with *KCNN2*-MDS.

|  | woman (mother) – P2 | | | girl (daughter) - P1 | | |
| --- | --- | --- | --- | --- | --- | --- |
| Age at testing | 38 years, 8 months | | | 17 years, 7 months | | |
| Years of education | 12 | | | 8 | | |
| handedness | right-handed | | | left-handed | | |
| Neuropsychiatric diagnoses and important psychiatric symptoms | schizophrenia;  obsessive compulsive symptoms;  depressed mood, suicidal attempts | | | past: anorexia nervosa; current: food fads  emotional lability, irritability  obsessive-compulsive symptoms  visual, tactile and olfactory hallucinations | | |
| Predominant neuropsychological profile | memory and executive dysfunction | | | memory and executive dysfunction | | |
|  | **raw score** | **standardized score^1^ [with CI] / scaled score^2^ / (per)centile^3 /^ T score^4 /^ sten score^5^** | **interpretation** | **raw score** | **standardized score^1^ [with CI] / scaled score^2^ / (per)centile^3 /^ T score^4 /^ sten score^5^** | **interpretation** |
| **General intellectual functioning**  Raven’s 2 Progressive Matrices D-E  Abstract thinking – Similarities from WAIS-R | 454  14 | 70  [59,81]^1^  9^2^ | low score  average score | 522  14 | 98  [85,107]^1^  10^2^ | average score  average score |
| **Cognitive screening**  DRS-2: total score / 144  attention / 37  initiation / perseveration / 37  construction / 6  conceptualization / 39  memory / 25 | 132  36  35  6  34  21 | 7^3*^  11^3*^  8^3*^  10^3*^  8^3*^  6^3*^ | above cut-off  average score  average score  average score  average score  low score | 136  37  35  6  39  19 | 9^3*^  13^3*^  8^3*^  10^3*^  12^3*^  4^3*^ | above cut-off  average score  average score  average score  average score  low score |
| **Manual dexterity and praxis**  9 Hole Peg test – right hand   - left hand   Grooved pegboard – right hand   - left hand   Spatial praxis  – interlocking fingers test / 4  Efferent motor praxis   - first sequence / 5 - second sequence / 5 | 28s.  27s.  124s.  139s.  2  0  0 |  | - 5SD  - 3SD  - 7SD  - 4,5 SD  impaired  impaired  impaired | 28s.  22s.  79s.  98s.  4  5  5 |  | -3SD (non-dominant)  -2 SD (dominant)  - 2SD (non-dominant)  - 1SD (dominant)  preserved  preserved (left hand)  preserved (left hand) |
| **Processing speed**  Digit Symbol from WAIS-R | 29 | 6^2^ | low score | 42 | 7^2^ | below average |
| **Language function**  CSB-Naming / 64  Semantic fluency – animals / fruit  Comprehension: Token test – short version / 36 | 63  17 / 17  28,5 | -  4^5^ / 5^5^  ^-^ | preserved  average  impaired | 63  15 / 18  36 | -  3 / 5  - | preserved  below expected***  preserved |
| **Visuospatial function**  VOSP Incomplete letters / 20  VOSP Dot counting /10  VOSP Cube analysis / 10  JoLO / 30 | 18  9  5  - | above cut-off  above cut-off  below cut-off | preserved  preserved  impaired | 20  10  10  22 | above cut-off  above cut-off  above cut-off  22^3**^ | preserved  preserved  preserved  low average |
| **Working memory**  months forward  months backwards  TMT A  TMT B  Digit Span – forward / backwards (max.)  Digit Span – total score  Corsi Block Tapping task:  max. span forward / max. span backwards  Serial sevens (max. 14) | 12; 5s.  10; 28s.  49s.  162s.  5 / 3  8  -  -  - | -  -  52^4^  55^4^  -  8^2^ | impaired  average  average  impaired  average | 30  64  5 / 4  9  5 / 5  13; 69s. | -  -  45^4^  45^4^  -  9^2^  -  - | average  average  average  preserved  slow |
| **Calculation**  WAB – multiple choice / 24 | 22 |  | impaired | - |  |  |
| **Episodic memory and learning**  CVLT – learning curve  trials 1-5 total  list B  short-delay free recall  short-delay cued recall  long-delay free recall  long-delay cued recall  intrusions in free recall  intrusions in cued recall  recognition: hits / false alarms  total recognition discrimination  CVMT – total score / 91   - delayed recognition task/ 7 - visual discrimination task / 7 | 4-7-7-9-9  37  3  3  4  3  3  8  4  6 / 1  75  65  1  5 | 30^4^  2^5^  1^5^  1^5^  1^5^  1^5^  2^5^  3^5^  1^5^ / 4^5^  1^5^  1,4^3^  1,4^3^ | impaired  impaired  impaired  impaired  impaired  impaired  impaired  impaired  impaired  impaired  impaired; below chance  impaired | 4-4-5-8-8  29  3  6  8  5  6  1  1  9 / 2  79,5  68  5  7 | -  22^4^  2^5^  1^5^  2^5^  1^5^  1^5^  6 ^5^  5^5^  1^5^ / 3^5^  1^5^  <1,2^3^  48,2^3^ | impaired  impaired  impaired  impaired  impaired  impaired  average score  average score  impaired  impaired  impaired  preserved  intact |
| **Executive function**  FAB / 18  Phonemic fluency – K / P / S / M  Cognitive flexibility:  WCST – categories achieved  WCST – total errors  WCST – conceptual level responses  Weigl Block Sorting Task / 9  Picture sequencing task / 3  Planning and cognitive control  ToL-DX2 – total move score  TOL-DX2 – total correct score  ToL-DX2 – rule violations  Cognitive control  Stroop interference test:  - color naming / 45s.  -color reading / 45s.  -interference / 45s. | 13  18 / 15 / 9 / 15  0  96  5  9  1  73  1  3  55  84  32 | -  7^5^ /- /- /-  6-10^3^  68^1^  65^1^  ≤60^1^  78^1^  ≤60^1^ | impaired  average  impaired  impaired  impaired  preserved  impaired  impaired  impaired  impaired  slowed performance | 18  5 / 6 / 12 / 2  6  10  83  -  -  55  2  0  60  80  45 | -  1^5^ /- /- /-  >16^3^  127^1^  112^1^  72^1^  86^1^  104^1^  - | above cut-off  impaired***  normal range  superior level  high average  borderline  low average  average  slowed  performance |
| **Depressive symptoms**  BDI-II | 54 | 10^5^ | severely depressed mood | 8 | 6^5^ | average score |

*based on a group aged 56-68 years, as there is no normative data for younger individuals

** based on a group aged 56-62 years, as there is no normative data for younger individuals

*** based on a group aged 18-39

BDI, Beck Depression Inventory; CI, confidence intervals; CSB, Cambridge Semantic Battery; CVLT, California Verbal Learning Test; CVMT, Continuous Visual Memory Test; DRS, Dementia Rating Scale; FAB, Frontal Assessment Battery; JoLO, Benton Judgement of Line Orientation Test; TOL-DX2, Tower of London Drexel University 2^nd^ edition; TMT, Trail Making Test; VOSP, Visual Object and Spatial Perception Test; WAB, Western Aphasia Battery; WAIS-R, Wechsler Adult Intelligence Scale – Revised; WCST, Wisconsin Card Sorting Test

Figure S1. Electrophysiological tests in P1 and P3 subjects.

**A)** In P1, EMG with a concentric needle electrode showed abnormal spontaneous activity in the first interosseus dorsalis muscle (IOD), with bursts from 100ms to 200ms duration. **B)** In P1, a 16Hz tremor was observed. **C)** In P3, resting EMG of the deltoid muscle revealed irregular myoclonic bursts lasting from 45ms to 80ms.


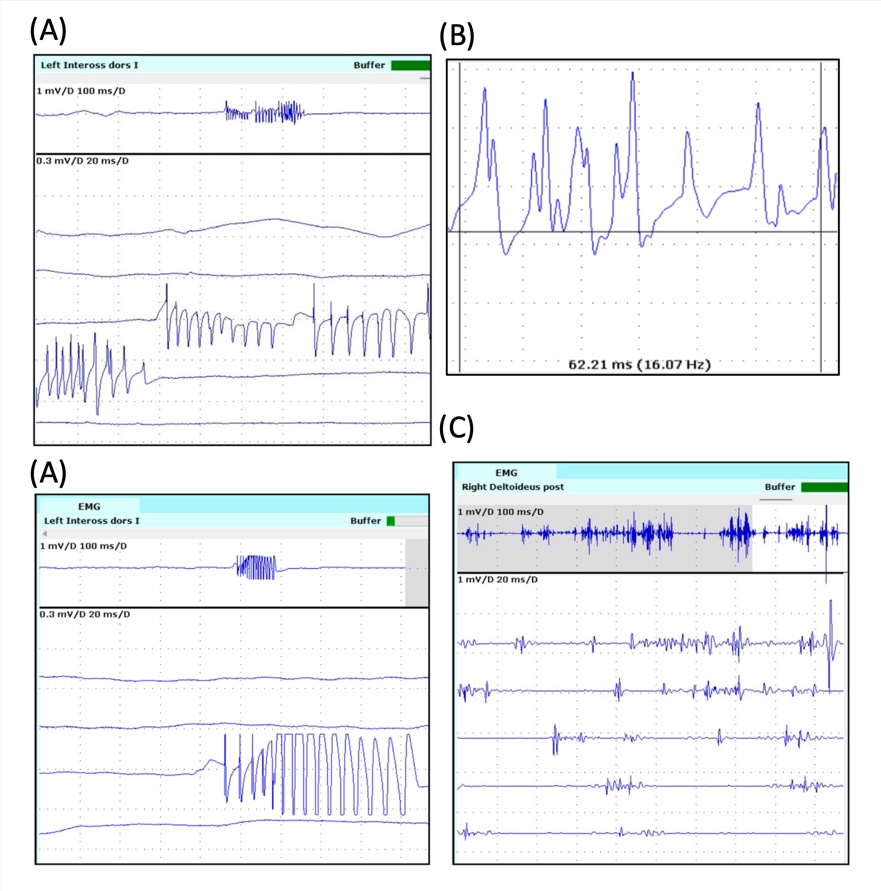

Supplement: Supplementary file 4 [file supplementary_file_1.docx]
